# Cardiac Arrest after Connecting Negative Pressure to the Subgaleal Drain during Craniotomy Closure

**DOI:** 10.1155/2014/146870

**Published:** 2014-05-22

**Authors:** Monu Yadav, Sapna A. Nikhar, Dilip Kumar Kulkarni, R. Gopinath

**Affiliations:** Department of Anaesthesiology & Critical Care, Nizam's Institute of Medical Sciences, Hyderabad 500082, India

## Abstract

A one-year-old child operated on for arachnoid cyst in right frontoparietotemporal region had sudden bradycardia followed by cardiac arrest leading to death after connecting negative pressure to the subgaleal drain during craniotomy closure. The surgical procedure was uneventful. It is a common practice to place epidural or subgaleal drains connected to a vacuum system towards the end of craniotomy to prevent accumulation of intracranial and extracranial blood. The phenomenon of bradycardia with hypotension is known to occur following negative pressure application to the epidural, epicranial, or subgaleal space after craniotomy closure. However cardiac arrest as a complication of negative pressure suction drain in neurosurgical patients is not described in the literature.

## 1. Introduction

It is a routine practice to put subgaleal drain during craniotomy closure after intracranial surgeries. It allows evacuation of collected blood and prevents hematoma formation and its consequences. Though it appears safe, it has its own risks and wide range of cardiovascular complications, from bradycardia and hypotension to even cardiac asystole. Application of sudden negative pressure to subgaleal drain should always be avoided. Anesthetist should also be very vigilant and should continue monitoring till the end of procedure.

## 2. Case Description

A one-year-old child was admitted with complaints of weakness of left upper limb and lower limb as noted by the parents for six months. He also had a history of seizures for the same duration. Child was born at full term by lower segment caesarean section with no history of any birth asphyxia. He was investigated; all his laboratory investigations were within normal limits. But his MRI (magnetic resonance imaging) revealed arachnoid cyst in right frontoparietotemporal region (8.2 × 6.2 × 4 cm) causing mild compression over adjacent brain parenchyma with architectural distortion and bilateral frontotemporal cortical atrophy.

Child was operated on and craniotomy with excision of arachnoid cyst was done under general anesthesia with controlled ventilation. Intraop monitoring included ECG, heart rate, noninvasive blood pressure, SpO_2_ (oxygen saturation), ETCO_2_ (end tidal CO_2_), urine output, and blood loss. Induction of anesthesia and intraoperative periods were uneventful. Arachnoid cyst was marsupialised by double fenestration followed by partial excision of cyst wall. At the end of the surgery on table extubation was planned. As a routine practice subgaleal drain was put during closure. Immediately after the drain was connected to the negative pressure system the child had sudden bradycardia followed by cardiac arrest. Immediately cardiopulmonary resuscitation started. Child was revived to normal sinus rhythm with heart rate of 110/min and blood pressure of 80/50 mmhg with dopamine iv infusion 2 mL/hr (12.5 mg/50 mL) and was shifted to neurosurgical intensive care unit for elective ventilation. On the first postoperative day CT (computerised tomography) of the brain revealed postcraniotomy defect in right frontoparietal lobe, parenchymal loss with large CSF attenuating collection adjacent to right sylvian fissure with internal hyperdensity, and likely porencephalic cyst with internal bleed with intraventricular extension.

The next two days he showed improvement in his respiratory parameters and was gradually weaned from ventilator support and was put on to T piece. Rate of dopamine infusion gradually tapered off to 1 mL/hr and then stopped. But neurologically the child did not show any improvement. On fifth postoperative day MRI of brain was repeated again which revealed an increase in the internal bleed in pore cephalic cyst with the rest of the findings similar to the previous MRI. On the same day child started deteriorating again and was put on ventilator. He developed bradycardia and hypotension which did not respond to any resuscitative measures and progressed to sudden cardiac arrest leading to death.

## 3. Discussion

Although there are several reports available describing hemodynamic disturbances such as bradycardia and hypotension following placement of negative pressure suction to epidural, epicranial, or subgaleal drain after neurosurgical procedure, only a few case reports are available on cardiac arrest as a complication of negative pressure suction drain in neurosurgical patients. Hernández-Palazón et al. [1] described severe bradycardia and reduction in intracranial pressure with the application of negative pressure through the epidural drainage connected to a vacuum system. But they did not report occurrence of any hypotension in their report. However, there is no case report existing describing sudden bradycardia followed by cardiac arrest in a paediatric patient. Although Bhagat et al. [2] reported sudden asystole in two adult neurosurgical patients during application of negative pressure suction to the subgaleal drain, they described that the patient responded to disconnection of the negative pressure to subgaleal space and on reconnection there was only bradycardia. The proposed mechanisms in these cases were sudden intracranial hypotension or trigeminocardiac reflex.

The trigeminocardiac reflex (TCR) is defined as the sudden onset of parasympathetic activity, sympathetic hypotension, apnoea, or gastric hypermotility during central or peripheral stimulation of any of the sensory branches of the trigeminal nerve. It has been reported to occur during craniofacial surgery, during manipulation of the trigeminal nerve/ganglion, and during surgery for lesion in the cerebellopontine angle, cavernous sinus, and the pituitary fossa. The manifestation of the TCR can vary from bradycardia and hypotension to asystole.

They also reported possibility of reflex fatigue on repetitive stimulation resulting in less severe hemodynamic derangements. The phenomenon of pseudohypoxic brain swelling (PHBS) was considered unlikely as it was an acute event without any delay in postoperative recovery.

PHBS is a severe and sometimes fatal complication after uneventful intracranial surgery. The clinical presentation and imaging features mimic those of global cerebral hypoxia. PHBS can occur in a mild, moderate, or severe degree. It is characterized by a very early postoperative onset of clinical deterioration (clouded or lost consciousness and pupillary abnormalities), in association with typical bilateral computed tomography or magnetic resonance imaging changes (hypodensities or altered intensities in the basal ganglia and/or thalamus).

Karamchandani et al. [3] also reported three episodes of bradycardia and hypotension after connecting vacuum drainage to subgaleal drain after uneventful surgery for cerebral aneurysm in a patient who had no significant cardiac history. Patient responded to atropine injection and after antagonization of neuromuscular block the patient recovered well. Toshniwal et al. [4] also reported a case of bradycardia following negative pressure suction of subgaleal drain; they also reported that there was a temporal association between the intensity of negative pressure applied and the time of occurrence of bradycardia.

In another case report Prabhakar et al. [5] mentioned rupture of intracranial aneurysm after partial clipping, with the use of the vacuum drain after an elective aneurysmal clipping surgery. They attributed it to the sudden negative pressure drainage leading to intracranial hypotension.

Although the exact mechanism of these serious hemodynamic changes ranging from bradycardia to even sudden cardiac arrest on application of negative pressure to subgaleal drain is yet to be found, it seems to be very important that negative pressure to the drain be applied very gradually. Sudden negative pressure should always be avoided. Keeping the possibilities of such serious complications in mind it is important for the anesthesiologist to be very vigilant during the craniotomy closure especially at time of connecting the drain to the negative pressure suction system. Close monitoring of the patient should even be continued towards the end of neurosurgical procedure.
